# Comparative Carcinogenicity of Double-Walled Carbon Nanotubes of Different Lengths Administered by Intratracheal Installation into Rat Lungs

**DOI:** 10.3390/nano15181402

**Published:** 2025-09-11

**Authors:** Omnia Hosny Mohamed Ahmed, Dina Mourad Saleh, William T. Alexander, Hiroshi Takase, Yuhji Taquahashi, Motoki Hojo, Ai Maeno, Katsumi Fukamachi, Min Gi, Akihiko Hirose, Shuji Tsuruoka, Satoru Takahashi, Hiroyuki Tsuda, Aya Naiki-Ito

**Affiliations:** 1 Nanotoxicology Project, Nagoya City University, Nagoya 467-8603, Japan; drdina@aun.edu.eg (D.M.S.); william@phar.nagoya-cu.ac.jp (W.T.A.); htsuda@phar.nagoya-cu.ac.jp (H.T.); 2 Department of Experimental Pathology and Tumor Biology, Graduate School of Medical Sciences, Nagoya City University, Nagoya 461-8601, Japan; sattak@med.nagoya-cu.ac.jp; 3 Department of Forensic Medicine and Clinical Toxicology, Faculty of Medicine, Aswan University, Aswan 81528, Egypt; 4 Department of Forensic Medicine and Clinical Toxicology, Faculty of Medicine, Assiut University, Assiut 71515, Egypt; 5 Core Laboratory, Graduate School of Medical Sciences, Nagoya City University, Nagoya 461-8601, Japan; takase@med.nagoya-cu.ac.jp; 6 Division of Cellular and Molecular Toxicology, Center for Biological Safety & Research, National Institute of Health Sciences, Kawasaki 210-0821, Japan; taquahashi@nihs.go.jp; 7 Department of Pharmaceutical and Environmental Sciences, Tokyo Metropolitan Institute of Public Health, Tokyo 169-0073, Japan; 8 Department of Neurotoxicology, Graduate School of Medical Sciences, Nagoya City University, Nagoya 461-8601, Japan; kfukamac@med.nagoya-cu.ac.jp; 9 Department of Environmental Risk Assessment, Graduate School of Medicine, Osaka Metropolitan University, Osaka 545-8585, Japan; mwei@omu.ac.jp; 10 Chemicals Evaluation and Research Institute (CERI), Tokyo 112-0004, Japan; akihikoh@dranihs.net; 11 Research Institute for Supra-Materials, Shinshu University, Nagano 380-8553, Japan; sh.tsuruoka@neura.co.jp

**Keywords:** carbon nanotubes, DWCNT, lung carcinogenicity, rats

## Abstract

We previously carried out an in vivo 2-year study to assess the potential toxicity/carcinogenicity of double-walled carbon nanotubes (DWCNTs) in a rat lung. We found that administration of DWCNTs by intratracheal–intrapulmonary spraying (TIPS) at a dose of 0.5 mg/rat induced the development of lung tumors in 7 of 24 treated rats while 1 of 21 untreated rats and 1 of 25 vehicle treated rats developed lung tumors. In the current study, we administered DWCNTs of different lengths, 1.5 µm, 7 µm, and 15 µm, to rats by TIPS to investigate the possible effect of the length of this thin, flexible CNT on toxicity/carcinogenicity in rat lungs. Rats were administered DWCNTs with lengths of 1.5 µm (D1.5), 7 µm (D7), and 15 µm (D15) by TIPS once every other day over the course of two weeks for a total of eight administrations. The total dose administered was approximately 22 × 10^12^ fibers per rat, corresponding to 0.0504 mg for D1.5, 0.232 mg for D7, and 0.504 mg for D15. Another group of rats was administered 0.5 mg MWCNT-7, a known carcinogen. Animals were killed at weeks 6 and 104 (4 and 102 weeks after the final TIPS administration). The mean survival time of the rats in the untreated, vehicle, D1.5, D7, and D15 groups was 99 to 104 weeks. One rat in the D1.5 group and one rat in the D15 group died before week 75. The remaining rats in the untreated, vehicle, D1.5, D7, and D15 groups were included in the final assessment of lung toxicity/carcinogenicity. In contrast, 11 rats in the MWCNT-7 group died before week 75 due to the development of malignant mesothelioma. Due to the much shorter survival time of the rats treated with MWCNT-7, accurate assessment of lung proliferative lesions in this group was not possible. At week 6, an increase in alveolar macrophages and granulation tissue foci in the alveoli was observed in all DWCNT administered groups. The alveolar epithelial cell PCNA index was also significantly increased in the D7 and D15 groups. Increases in alveolar macrophages, granulation tissue foci, and the alveolar epithelial cell PCNA index were observed in all DWCNT-treated groups at the final sacrifice. The incidences of lung tumors were 0/13, 0/12, 4/12, 3/8, and 2/10 in the untreated, vehicle, D1.5, D7, and D15 groups, respectively. In agreement with our previous study, the DWCNTs tested in the present study were carcinogenic in the rat lung. In addition, we present evidence that DWCNT fiber length may possibly have an effect on DWCNT-induced carcinogenicity in rat lungs.

## 1. Introduction

Carbon nanotubes (CNTs) are extremely valuable materials with a wide range of applications. However, because of their light weight, individual CNTs and small CNT agglomerates are easily airborne and inhaled. Despite the widespread production and use of carbon nanotubes (CNTs), long-term in vivo studies to examine the toxicity and carcinogenicity of CNTs in the lung are extremely limited. To date, only eleven long-term lung carcinogenicity studies have been reported: Sargent et al. (2014) [1], Fujita et al. (2015) [2], Suzui et al. (2016) [3], Kasai et al. (2016) [4], Honda et al. (2017) [5], Numano et al. (2019) [6], Saleh et al. (2020) [7], Hojo et al. (2022) [8], Saleh et al. (2022) [9], Sheema et al. (2024) [10]. Consequently, little is known about the chronic lung toxicity/carcinogenicity of the vast majority of CNTs.

Initial studies of CNTs administered the fibers by intraperitoneal and intrascrotal injection, as reviewed by Ahmed et al. (2025) [11]. These studies found that thicker, rigid CNTs with multiple wall layers (MWCNTs) induced the development of mesotheliomas, while thinner, flexible MWCNTs did not induce the development of cancers. The conclusion of these studies was that thin, flexible CNTs are not carcinogenic. However, our research challenges this assumption. In a two-year inhalation study using intratracheal–intrapulmonary spraying (TIPS), we demonstrated that a thin, flexible MWCNT (with 15 wall layers) was carcinogenic in rat lungs [7]. The carcinogenicity of this thin, flexible MWCNT indicated that intraperitoneal injection of fibers does not necessarily specify the carcinogenicity of the fibers when they are inhaled into the lung. We subsequently conducted a similar two-year TIPS study using double-walled carbon nanotubes (DWCNTs), which consist of only two wall layers. These DWCNTs also induced lung tumors in rats [9], further underscoring the importance of evaluating fiber toxicity in a respiratory context.

The mechanisms of CNT-induced carcinogenesis appear to differ between anatomical sites. In the peritoneal cavity, thick, rigid CNTs physically damage mesothelial cells, triggering the release of HMGB1 and promoting mesothelioma development [9,12,13,14]. Thin, flexible CNTs, in contrast, do not cause such cellular injury and are less likely to provoke cancer [9,15,16]. In the lung, however, CNTs interact with alveolar macrophages, initiating cytokine-mediated inflammation that can progress to fibrosis and tumorigenesis [11].

Given the established pulmonary carcinogenicity of DWCNTs [9], the current study aimed to explore whether CNT fiber length influences lung toxicity and carcinogenicity. Using the TIPS method, we exposed rats to DWCNTs of three different lengths: 1.5 μm (D1.5), 7 μm (D7), and 15 μm (D15). All three groups developed lung tumors. While tumor incidence did not vary significantly among the groups, consideration of bronchoalveolar hyperplasia suggests that fiber length may modulate carcinogenic potential.

## 2. Materials and Methods

### 2.1. DWCNTs and MWCNT-7

DWCNTs of 3 different lengths, 1.5, 7, and 15 µm, were supplied by Neura Inc., Tokyo, Japan. These DWCNTs contained iron levels below the detectable limit. MWCNT-7 (40–50 wall layers, 66.8 nm mean diameter, 6.65 μm mean length) was supplied by Mitsui Chemicals Inc. (Tokyo, Japan). MWCNT-7 had an iron content of 0.3% by weight [16].

### 2.2. CNT Suspensions

DWCNT 1.5 µm in length (D1.5): 1.008 mg of D1.5 fibers with a total fiber count of 4.256 × 10^14^ was suspended in 80 mL saline containing 0.5% poloxamer 188 solution (P5556; Sigma-Aldrich, St. Louis, MO, USA), yielding 6.3 µg/2.660 × 10^12^ fibers per 500 µL saline solution. DWCNT 7 µm in length (D7): 4.066 mg of D7 fibers with a total fiber count of 3.808 × 10^14^ was suspended in 70 mL saline containing 0.5% poloxamer 188 solution, yielding 29.0 µg/2.720 × 10^12^ fibers per 500 µL saline solution. DWCNT 15 µm in length (D15): 8.160 mg of D15 fibers with a total fiber count of 3.584 × 10^14^ was suspended in 64.75 mL saline containing 0.5% poloxamer 188 solution, yielding 63.0 µg/2.767 × 10^12^ fibers per 500 µL saline solution. MWCNT-7 was suspended in saline containing 0.5% poloxamer 188 solution at a concentration of 0.126 mg/mL.

### 2.3. Animals

Ten-week-old male F344/rats (F344/DuCrlCrlj) were purchased from Charles River Laboratories Japan, Inc., Kanagawa, Japan. Animals were housed in the Center for Experimental Animal Science of Nagoya City University Medical School, maintained on a 12/12 h light/dark cycle, and received an Oriental MF Basal diet (Oriental Yeast, Tokyo, Japan) and tap water ad libitum. The study was conducted according to the Guidelines for the Care and Use of Laboratory Animals of Nagoya City University Medical School (Nagoya, Japan), and the experimental protocol was approved by the Nagoya City University Animal Care and Use Committee.

### 2.4. Experimental Design

After acclimatization for 2 weeks, the rats were divided into 6 groups of 16–20 animals each: Group 1—untreated, 18 rats; Group 2—vehicle, 18 rats; Group 3, D1.5, 18 rats; Group 4, D7, 16 rats; Group 5, D15, 16 rats; Group 6, MWCNT-7, 20 rats. One rat in the vehicle group and 3 rats in the D7 group died during weeks 29–30 due to mechanical failure of the drinking water supply system and were removed from the study; therefore, the vehicle group was composed of 17 rats, and the D7 group was composed of 13 rats.

Immediately prior to each TIPS administration, the solutions containing the test materials were sonicated for 30 min, as previously described [9]. We confirmed using scanning electron microscopy (VE-9800, KEYENSE, Osaka, Japan) that the length of the DWCNTs was not affected by sonication.

Rats, under 3% isoflurane anesthesia, were administered the test materials in 500 µL saline containing 0.5% poloxamer 188 solution using TIPS once every other day over a 2-week period (total 8 doses). The single administration doses were 6.3 µg/2.660 × 10^12^ fibers of D1.5; 29.0 µg/2.720 × 10^12^ fibers of D7; 63.0 µg/2.767 × 10^12^ fibers of D15; and 63.0 µg of MWCNT-7. Total doses were 50.4 µg/2.13 × 10^13^ fibers per rat of D1.5; 232.3 µg/2.18 × 10^13^ fibers per rat of D7; 504.1 µg/2.21 × 10^13^ fibers per rat of D15; and 504.0 µg per rat of MWCNT-7.

At six weeks (4 weeks after the final TIPS administration), 5 rats per group underwent complete necropsy and histopathological examination to evaluate acute pulmonary effects. All rats that lived longer than 52 weeks were included in the assessment of mesothelioma development, and all rats that lived longer than 75 weeks were included in the assessment of chronic lung toxicity/carcinogenicity. All rats that were found in a moribund condition or found dead before the end of the study period at 104 weeks underwent complete necropsy and histopathological examination.

A total of 11 of the 15 rats administered MWCNT-7 died before 75 weeks due to the development of malignant mesothelioma. To relieve the remaining 4 rats of unnecessary suffering, these rats were sacrificed at week 75.

### 2.5. Tissue Sampling and Histopathology

The rats were deeply anesthetized by 5% isoflurane then killed by exsanguination from the inferior vena cava. The whole lung was excised, and the posterior lobe of right lung was tied off, and 10 mL of phosphate-buffered 4% paraformaldehyde solution was infused into the lung. The posterior lobe was frozen, and the remaining lobes were processed for histological analysis. The chest wall, major organs, and mediastinal and mesenteric lymph nodes were excised and fixed in phosphate-buffered 4% paraformaldehyde solution and processed for histological analysis. Identification of DWCNTs and MWCNT-7 in the lung and other tissue was confirmed using a microscope equipped with a polarizing lens (PLM; SX51N-31P-O, Olympus, Tokyo, Japan and N11599BNZ, KEYENCE, Osaka, Japan). Diagnosis of the induced lesions was performed by Hiroyuki Tsuda, M.D., Ph.D., a board-certified pathologist of the Japanese Society of Toxicologic Pathology (Diplomate of JSTP) and the Japanese Society of Pathology. Diagnosis is consistent with the INHAND project [17]; see pages 39S–46S for a discussion of the diagnosis of hyperplasia, adenoma, and carcinoma.

The total number of granulomas and CD68-positive macrophages were counted manually within sections from the entire 4 lobes from the fixed lung tissue. Total lung areas were determined using an image analyzer (KEYENCE BZ-X810). The frequency of granulomas and CD68-positive macrophages was determined by dividing their total numbers by the total area of the lung tissue examined. Data are presented as count/cm^2^.

For the PCNA index, sections were incubated with anti-PCNA (Cell Signaling Technology, Danvers, MA, USA) in concentration of 1:2000. In each section, more than 1000 pulmonary epithelial cells and more than 500 visceral and parietal pleural mesothelial cells were counted blindly in random fields. All nuclei showing brown staining of more than half of the nucleus were considered to be positive.

### 2.6. Electron Microscopy

The DWCNTs and MWCNT-7 test solutions used for TIPS administration were treated with ultrasound for 30 min, followed by placement on carbon supported film copper grid, and then observed by transmission electron microscopy (TEM) (JEM-1400Plus, JEOL, Tokyo, Japan).

To observe CNTs present in the lung alveoli, H&E-stained slides prepared from paraffin embedded blocks were immersed in xylene to remove the cover glass, dried, coated with a layer of sublimated OsO4 using an osmium plasma coater (OPC80N; Filgen, Nagoya, Japan), and then processed for scanning electron microscopy (SEM) (S-4800, Hitachi, Tokyo, Japan).

### 2.7. CCL2, CCL3, and HO-1 ELISA

Frozen non-tumor lung tissue samples (approximately 100 mg) were thawed and rinsed 3 times with ice-cold PBS and homogenized in 1 mL tissue protein extraction reagent (Thermo Scientific, Rockford, IL, USA) containing 1% (*v*/*v*) protease inhibitor cocktail (Sigma-Aldrich Merck, Darmstadt, Germany). The homogenates were centrifuged at 12,000× *g* for 5 min at 4 °C. The protein content of the supernatant was measured using the BCA Protein Assay Kit (Thermo Fischer Scientific, Massachusette, USA). The levels of CCL2, CCL3, and heme oxygenase 1 (HO-1) in the supernatant were measured using a Rat MCP-1/CCL2 ELISA Kit (Sigma-Aldrich Merck; RAB0058), a Rat CCL3 ELISA Kit (LSBio, Washington, USA, LS-F5526), and a Rat Heme Oxygenase 1 ELISA Kit (abcam, Cambridge, UK ab279414) according to the manufacturers’ instructions. Absorbance was measured using a microplate reader (SpectraMax iD3, Molecular Devices, San Jose, CA, USA) following the manufacturer’s instructions.

### 2.8. Measurement of DWCNT and MWCNT in the Lung

Measurement of the amount of CNT fibers in the lung tissue was performed as previously described [18].

### 2.9. Statistical Analysis

Statistical significance was analyzed using a 2-sided *t*-test with the GraphPad QuickCals T Test Calculator for continuous data and the Fisher exact test (one-tailed) for categorical data. Data are reported as mean ± SD. *p*-values < 0.05 were considered to be significant. The one-tailed Fisher exact test was used since there is no evidence that exposure to CNT fibers protects against the development of lung cancer.

## 3. Results

### 3.1. Characterization of Test CNTs

**Figure 1:**
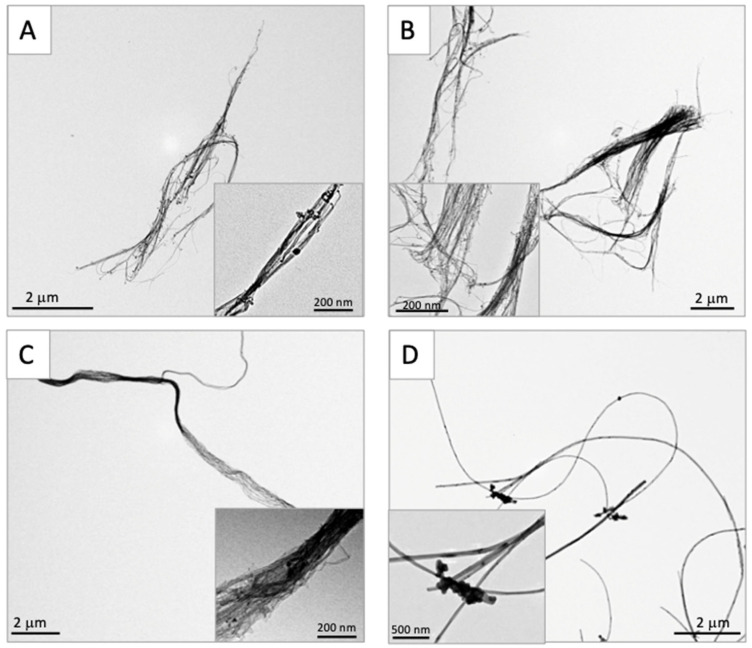
Transmission electron microscopic findings of three DWCNTs and MWCNT-7 before administration. (**A**) DWCNT (1.5 μm). (**B**) DWCNT (7 μm). (**C**) DWCNT (15 μm). (**D**) MWCNT-7. All the fibers were irregularly tangled in shape.

After the 30 min sonication of the test solutions prior to TIPS administration, 20 µL of the test solution was processed for TEM. Representative TEM images of the three different DWCNTs and MWCNT-7 in suspension, prior to TIPS administration, are shown in Figure 1. The three different DWCNTs were present as tangled agglomerates, agglomerates as defined by Walter, 2013 [19]. The sizes of the agglomerates are shown in Table 1.

### 3.2. Lung Lesions at Six Weeks (Table 2)

**Figure 2:**
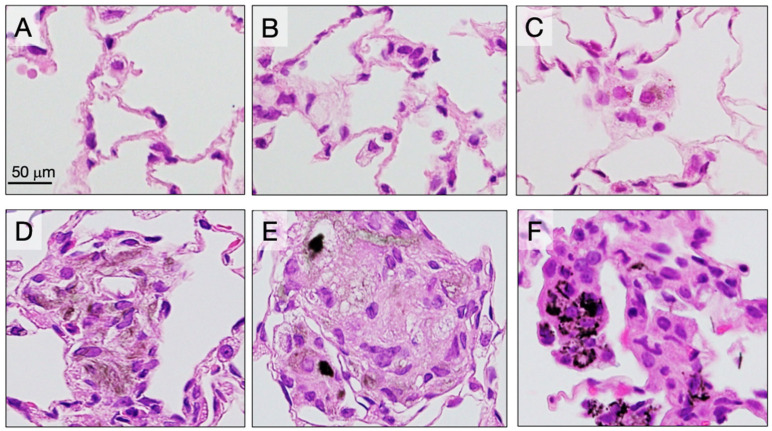
Granuloma formation in the alveoli at week 6. (**A**) Untreated. (**B**) Vehicle. (**C**) DWCNT 1.5 μm. (**D**) DWCNT 7 μm. (**E**) DWCNT 15 μm. (**F**) MWCNT-7. Lung histology sections of rats administered DWCNT and MWCNT-7 at 6 weeks. The majority of the DWCNT and MWCNT-7 are encapsulated in granulomas in the alveoli (**C**–**F**). Their fibers can be seen (brown and black areas) encapsulated inside the granulomas.

**Figure 3:**
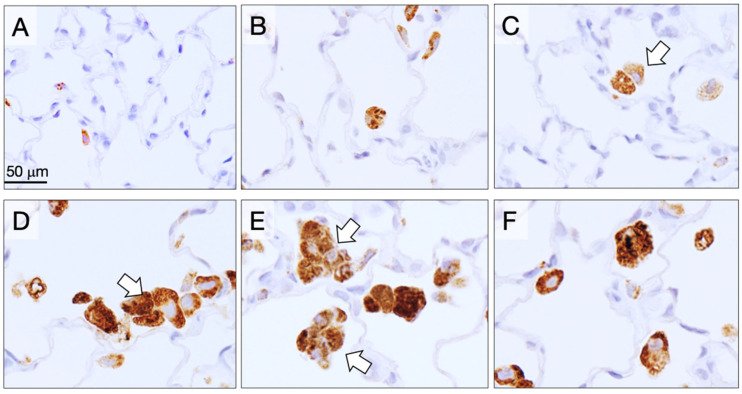
Alveolar CD68-positive macrophages (brown) at week 6. (**A**) Untreated. (**B**) Vehicle. (**C**) DWCNT 1.5 μm. (**D**) DWCNT 7 μm. (**E**) DWCNT 15 μm. (**F**) MWCNT-7. Lung sections of rats administered DWCNTs at 6 weeks. The majority of the DWCNT fibers are found in macrophages aggregates attached to the alveolar wall (arrows) (**C**–**E**).

Five rats from each group were sacrificed at 6 weeks (4 weeks after the final TIPS administration) to assess the acute lung toxicity of the CNTs. Granulomas were significantly increased in the lungs of rats treated with the three different DWCNTs and with MWCNT-7 (Figure 2). Notably, the number of granulomas in the DWCNT-treated rats increased with increasing DWCNT length. The number of CD68-positive macrophages in the alveoli was also significantly increased in rats treated with the three different DWCNTs and with MWCNT-7 (Figure 3). In contrast to granuloma count, the increase in macrophages was similar in the D1.5, D7, and D15 groups. Finally, alveolar epithelial cell PCNA count was significantly increased in rats treated with 7 μm and 15 μm DWCNT and MWCNT-7. PCNA count increased with increasing DWCNT length.

**Figure 4:**
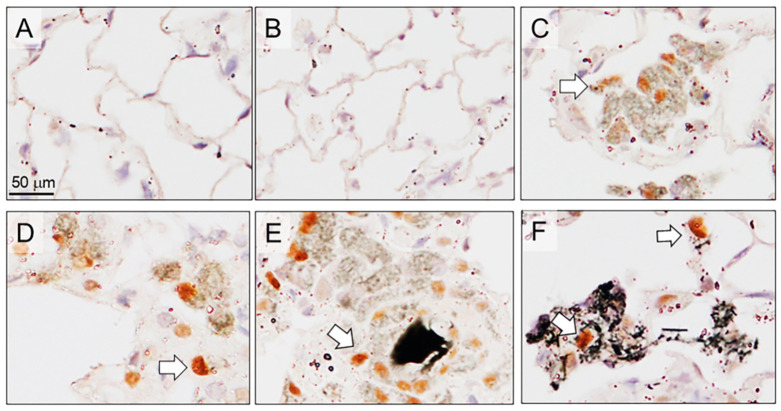
PCNA-positive macrophages (brown nuclei) in the alveoli at week 6. (**A**) Untreated. (**B**) Vehicle. (**C**) DWCNT 1.5 μm. (**D**) DWCNT 7 μm. (**E**) DWCNT 15 μm. (**F**) MWCNT-7. Lung sections of rats administered DWCNTs at 6 weeks. Among the PCNA-positive macrophages that compose the granulation tissue, some clearly phagocytose DWCNTs and MWCNT-7 (arrows) (**C**–**F**).

Macrophages forming granulation tissue engulfed DWCNTs and MWCNT-7, and most of these phagocytes were positive for PCNA (Figure 4). PCNA is also associated with DNA repair, and it has been shown that non-proliferating macrophages can be PCNA-positive [20]. This is consistent with the interaction of DWCNTs with macrophages inducing production reactive oxygen species (ROS) and other cytotoxic and DNA-damaging molecules by the macrophages in an attempt to destroy the alveoli-invading pathogens/fibers.

**Table 2:** Count of granulomas, macrophages, and alveolar epithelial cell proliferation at week 6 ^$^.

	Group	Group 1	Group 2	Group 3	Group 4	Group 5	Group 6
Tissue Parameter		Untreated	Vehicle	DWCNT			MWCNT-7 (0.5 ^#^)
				1.5 μm (0.050 ^#^)	7 μm (0.232 ^#^)	15 μm (0.504 ^#^)	
Granuloma count/cm^2^ Mean ± SD		0 ± 0	0.6 ± 0.5	12.1 ± 9.5 ***	50.5 ± 5.8 ***^a^	194.7 ± 36.2 ***^b^	49.8 ± 15.6 ***^c^
Macrophage cell ^&^ count/cm^2^ Mean ± SD		26.6 ± 19.4	124.3 ± 20.5	351.3 ± 26.6 ***	313.6 ± 15.5 ***	388.6 ± 29.3 ***^d^	468.6 ± 15.3 ***^e^
Alveolar epithelial cell PCNA index %		1.24 ± 0.55	0.93 ± 0.25	1.96 ± 0.23	3.73 ± 2.19 *	8.53 ± 3.63 ***	3.74 ± 0.79 ***

^$^ Five animals were examined in each group. ^#^ Total fiber dose (mg)/rat. ^&^ CD68-positive cells. *, *** *p* < 0.05, 0.001 vs. vehicle, respectively. ^a^ *p* < 0.01, DWCNT-7 μm vs. DWCNT-1.5 μm. ^b^ *p* < 0.01, DWCNT-15 μm vs. DWCNT-1.5 μm, DWCNT-7 μm and MWCNT-7. ^c^ *p* < 0.05, 0.01, MWCNT-7 vs. DWCNT-1.5 μm and DWCNT-15 μm, respectively. ^d^ *p* < 0.05, DWCNT-15 μm vs. DWCNT-7 μm. ^e^ *p* < 0.05, 0.01, 0.001, MWCNT-7 vs. DWCNT-15 μm, 1.5 μm, and 7 μm, respectively.

### 3.3. Survival Time

One rat in the vehicle control group and three rats in the D7 group died during weeks 29–30 due to mechanical failure of the drinking water supply system. The remaining rats in the D7 group lived to the end of the study period. One rat in the D1.5 group and one rat in the D15 group died before week 75. The mean survival times of the untreated, vehicle, D1.5, D7, and D15 groups were 99.1 ± 10.1, 101 ± 5.9, 102 ± 7.9, 104, and 100 ± 8.7 weeks, respectively. In contrast to these groups, 11 rats treated with MWCNT-7 died before week 75 due to the development of malignant mesothelioma.

### 3.4. Incidence of Mesothelioma (Table 3)

**Figure 5:**
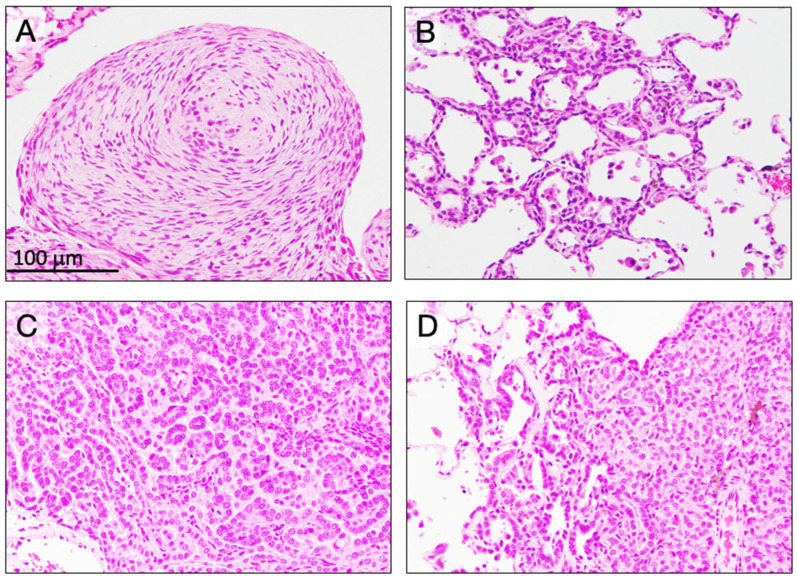
Proliferative lesions at the final sacrifice. (**A**) Malignant mesothelioma: concentric proliferation of spindle-shaped tumor cells on the surface of lung pleura and mediastinal tissue area. (**B**) Bronchioalveolar hyperplasia: bronchioalveolar cell are arranged in single layer, and the alveolar structure is not destroyed. (**C**) Bronchioloalveolar carcinoma: alveolar structure is destroyed by densely proliferating carcinoma cells. (**D**) Periphery of bronchioalveolar carcinoma showing invasion of surrounding alveoli.

All rats that survived for 52 weeks or longer were included in the assessment of pleural mesothelioma development. In the MWCNT-7 group, 11/15 rats developed pleural mesothelioma (Figure 5A) and died before week 75. As noted above, to relieve the remaining four rats of unnecessary suffering, these rats were sacrificed at week 75. One of these rats had also developed pleural mesothelioma. A malignant mesothelioma also developed on the visceral pleural surface of one rat in the D15 group. This rat was found dead and could not be assessed for the presence of HMGB1 in the pleural lavage. However, as discussed below, development of visceral pleural mesothelioma in rats is unlikely to be related to the development of pleural mesothelioma in humans. In addition, current evidence indicates that thin, flexible CNTs are not carcinogenic in the pleura, as reviewed by Ahmed et al. (2025) [11]. Therefore, the development of a single visceral mesothelioma in the D15 group is not evidence that D15 fibers administered into the lung induce pleural mesothelioma.

**Table 3:** Incidence of pleural mesothelioma at the final sacrifice ^$^.

	Group 1	Group 2	Group 3	Group 4	Group 5	Group 6
	Untreated	Vehicle	DWCNT			MWCNT-7
			1.5 μm (0.050 ^#^)	7 μm (0.232 ^#^)	15 μm (0.504 ^#^)	
No. of rats examined	13	12	13	8	11	15
Mean survival time (weeks)	99.1 ± 10.1	101 ± 5.9	102 ± 7.9	104	100 ± 8.7	74.9 ± 10.3
Pleural mesothelioma	0	0	0	0	1	12 ***

^$^ In addition to animals that lived to the end of the study, the final sacrifice includes all animals that lived longer than 52 weeks but were moribund or found dead before the end of the study period. ^#^ Total fiber dose (mg)/rat. Significantly different from vehicle at *p* < 0.001 (***), Fisher exact test.

### 3.5. Incidence of Preneoplastic and Neoplastic Lesions in the Lung (Table 4)

One rat in the D1.5 group and one rat in the D15 group died before week 75. All of the rats in the untreated, vehicle, and D7 groups lived longer than 75 weeks. All rats in the control and DWCNT groups that lived longer than 75 weeks were included in the final assessment of the development of preneoplastic and neoplastic lesions in the lung. Due to the early deaths of the MWCNT-7-treated rats, accurate assessment of MWCNT-7-induced lung proliferative lesions was not possible. Therefore, the MWCNT-7 group is not included in Table 4.

No hyperplastic or neoplastic bronchioloalveolar lesions were induced in the untreated or vehicle control groups. Development of bronchioloalveolar hyperplasia (Figure 5B) was found in five rats in the D7 group (*p* < 0.01) and five rats in the D15 group (*p* < 0.01). Bronchioloalveolar adenoma was not found in any group. Development of bronchioloalveolar carcinoma (Figure 5C,D) was found in four rats in the D1.5 group (*p* < 0.05), three rats in the D7 group (*p* < 0.05), and two rats in the D15 group. There were no statistically significant differences in tumor development between the D1.5, D7, and D15 groups.

While accurate assessment of MWCNT-7-induced lung proliferative lesions was not possible, the four rats that did not die from malignant mesothelioma before 75 weeks were examined for proliferative lung lesions. One rat developed bronchioloalveolar hyperplasia, and two rats developed bronchioloalveolar carcinoma. None of the four rats developed bronchioloalveolar adenoma.

**Table 4:** Incidence of lung proliferative lesions at the final sacrifice ^$^.

	Group 1	Group 2	Group 3	Group 4	Group 5
	Untreated	Vehicle	DWCNT		
			1.5 μm (0.050 ^#^)	7 μm (0.232 ^#^)	15 μm (0.504 ^#^)
No. of rats examined	13	12	12	8	10
Mean survival time (weeks)	99.1 ± 10.1	101 ± 5.9	102 ± 7.9	104	100 ± 8.7
Bronchioloalveolar lesions Hyperplasia (BAH)	0	0	0	5 **	5 **
Adenoma (BAA)	0	0	0	0	0
Carcinoma (BAC)	0	0	4 *	3 *	2

^$^ The final sacrifice also includes all animals that lived longer than 75 weeks but were moribund or found dead before the end of the study period. ^#^ Total fiber dose (mg)/rat. Significantly different from vehicle at *p* < 0.05 (*) and at *p* < 0.01 (**), Fisher exact test.

### 3.6. Fiber-Induced Toxicity Not Related to Pleural and Lung Toxicities

No clinical signs or toxicities that were not related to pleural and lung toxicities were elevated in the DWCNT-administered groups compared to the control groups.

### 3.7. Alveolar Granulomas, CD68-Positive Macrophages, and PCNA-Positive Lung Alveolar Epithelial and Pleural Cell Counts at the Final Sacrifice (Table 5)

**Figure 6:**
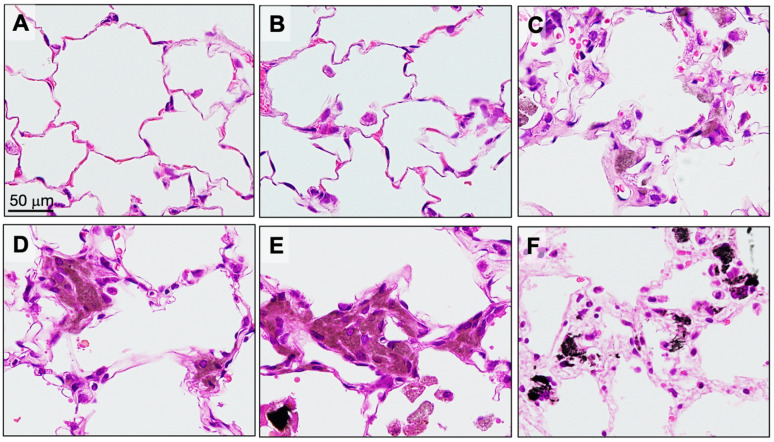
Engulfment of the three different-length DWCNTs and MWCNT-7 by alveolar macrophages at the final sacrifice. (**A**) Untreated. (**B**) Vehicle. (**C**) DWCNT 1.5 μm. (**D**) DWCNT 7 μm. (**E**) DWCNT 15 μm. (**F**) MWCNT-7. Lung H&E sections of rats administered three different-length DWCNTs and MWCNT-7 at the final sacrifice. Engulfed DWCNT fibers produce a brown color in the cytoplasm (**C**–**E**), and very large amounts of engulfed DWCNT fibers and MWCNT-7 fibers block penetration of the light, creating a black patch (**E**,**F**). Fibers are mostly engulfed by alveolar macrophages and encased in granulomas (**C**–**F**).

**Figure 7:**
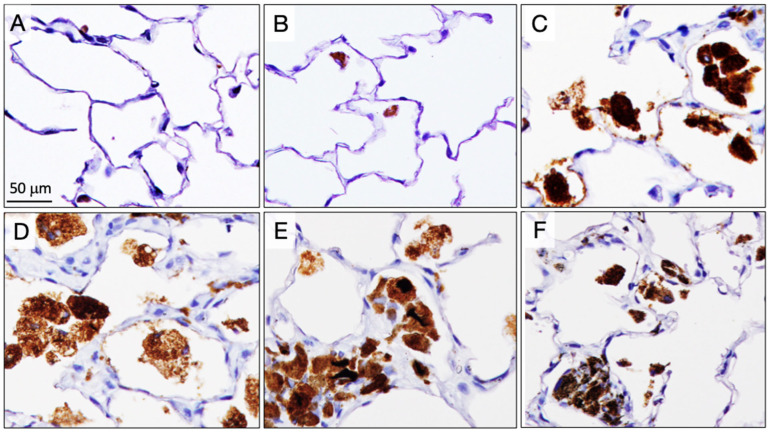
CD68-positive alveolar macrophages (brown) at the final sacrifice. (**A**) Untreated. (**B**) Vehicle. (**C**) DWCNT 1.5 μm. (**D**) DWCNT 7 μm. (**E**) DWCNT 15 μm. (**F**) MWCNT-7. DWCNTs were phagocytosed by macrophages in the alveoli or septal wall forming granulation tissue. This is clearly different from the 6-week finding that DWCNT engulfing macrophages are more abundant in the alveolar space.

**Figure 8:**
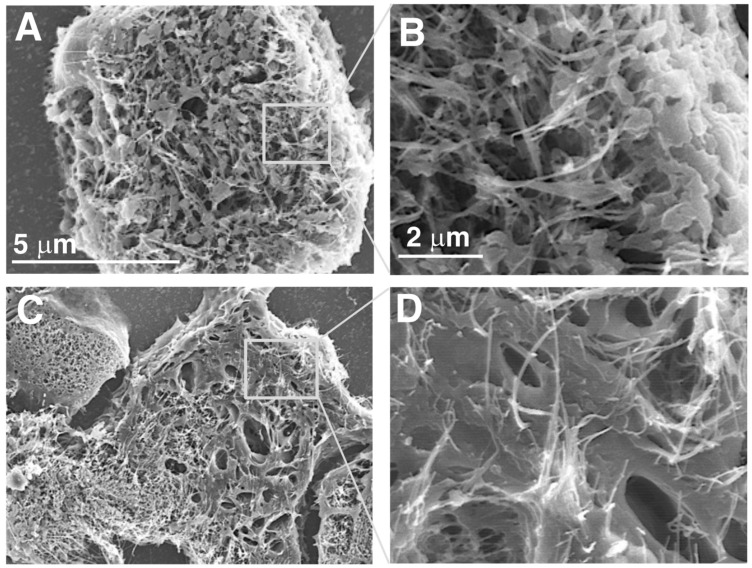

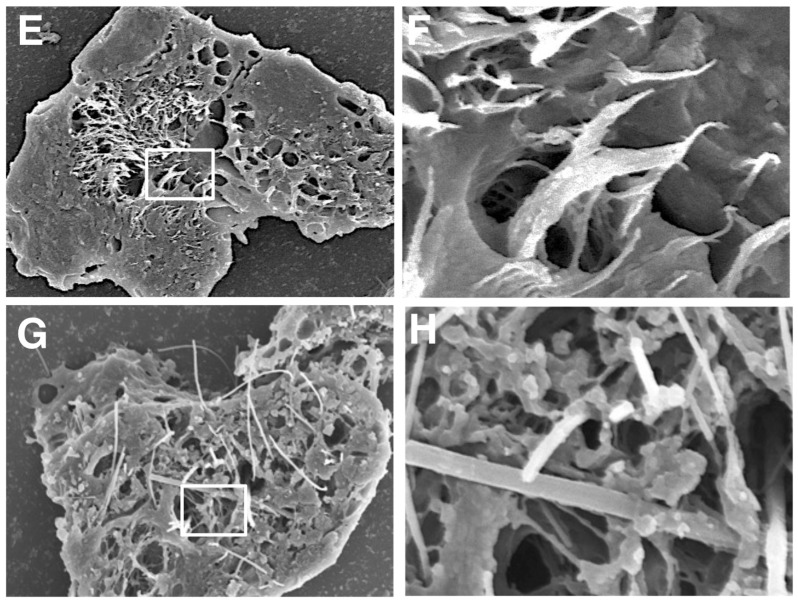
Scanning electron microscopic photos of DWCNTs engulfed by alveolar macrophages. (**A**) DWCNT 1.5 μm. (**B**) Higher magnification of (**A**). Fiber agglomerates are irregularly shaped and somewhat thinner than DWCNT 7 µm and DWCNT 15 µm. (**C**) DWCNT 7 µm. (**D**) Higher magnification of (**C**). Fiber agglomerates are thin like (**A**) but are partly flattened. (**E**) DWCNT 15 μm. (**F**) Higher magnification of (**E**). Fibers form irregular agglomerates with thin to thick or flat shapes. (**G**) MWCNT-7. (**H**) Higher magnification of (**G**). Fibers are needle/thread-shaped agglomerates, some stabbing through the cell membrane. Overall findings: no clear length-dependent related differences in the shape of the DWCNT agglomerates were observed.

Fibers in the alveoli were phagocytosed by macrophages, forming granulomas (Figure 6). Granuloma count was significantly increased in all DWCNT groups compared with untreated/vehicle controls, and the granuloma count increased with the increase in DWCNT fiber length. There was also a significant increase in CD68-positive alveolar macrophages (Figure 7) in the DWCNT groups compared with untreated/vehicle controls, and the CD68-positive macrophage count increased with the increase in DWCNT fiber length. Scanning electron microscopic observation of the macrophages indicates that alveolar macrophages had clearly engulfed DWCNT and MWCNT-7 fibers (Figure 8A–H).

**Figure 9:**
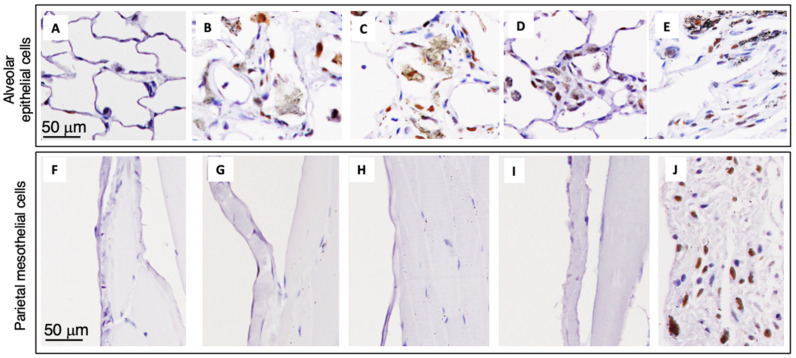
Proliferating cell nuclear antigen (PCNA) staining in the alveolar epithelial and parietal mesothelial cells from rats treated with DWCNT and MWCNT-7 at the final sacrifice. (**A**,**F**) Vehicle. (**B**,**G**) DWCNT 1.5 μm. (**C**,**H**) DWCNT 7 μm. (**D**,**I**) DWCNT 15 μm. (**E**,**J**) MWCNT-7.

Similarly to granuloma count and CD68-positive macrophages, there was a significant increase in PCNA labelling of alveolar epithelial cells in the DWCNT groups compared with untreated/vehicle controls, and PCNA labelling increased with the increase in DWCNT fiber length. In DWCNT- and MWCNT-7-treated rats, alveolar epithelial and some alveolar stromal cells were positive for PCNA, and many alveolar wall cells were also positive for PCNA (Figure 9A–E). In contrast, parietal tissue on the diaphragm (Figure 9F–I), the visceral pleura, and parietal pleura was negative for PCNA in the DWCNT-treated rats. However, in the MWCNT-7-treated rats, many parietal pleural cells were strongly positive for PCNA (Figure 9J). This is consistent with the development of mesothelioma in the MWCNT-7-treated rats.

**Table 5:** Alveolar granulomas, CD68-positive macrophages, and PCNA-positive alveolar epithelial and pleural mesothelial cell count at the final sacrifice ^$^.

	Group	Group 1	Group 2	Group 3	Group 4	Group 5	Group 6
Tissue Parameter		Untreated	Vehicle	DWCNT			MWCNT-7
				1.5 μm (0.050 ^#^)	7 μm (0.232 ^#^)	15 μm (0.504 ^#^)	
Granuloma count/cm^2^ Mean ± SD		1.0 ± 0.8	1.0 ± 0.8	43.7 ± 14.4 ***	133.7 ± 46.3 ***^a^	188.5 ± 71.2 ***^b^	83.8 ± 42.0 ***
Macrophage cell ^&^ count/cm^2^ Mean ± SD		78.5 ± 15.1	87.2 ± 16.2	250.4 ± 48.8 ***	323.6 ± 36.5 ***^c^	427.6 ± 96.8 ***^d^	224.4 ± 82.3 ***^e^
Alveolar epithelial cell PCNA index %		2.1 ± 0.1	1.9 ± 0.6	12.4 ± 4.5 **	18.7 ± 4.2 **	29.2 ± 9.8 ***^f^	24.5 ± 14.1 ***
Pleural mesothelial cell PCNA index %		0.4 ± 0.4	0.5 ± 0.2	0.8 ± 0.5	1.0 ± 0.6	0.9 ± 0.6	37.4 ± 12.5 ***^g^

^$^ Five animals were examined in each group. ^#^ Total fiber dose (mg)/rat. ^&^ CD68-positive cells. **, *** *p* < 0.01, 0.001 vs. vehicle, respectively. ^a^ *p* < 0.01, DWCNT-7 μm vs. DWCNT-1.5 μm. ^b^ *p* < 0.01, DWCNT-15 μm vs. DWCNT-1.5 μm and MWCNT-7. ^c^ *p* < 0.05, DWCNT-7 μm vs. DWCNT-1.5 μm. ^d^ *p* < 0.001, DWCNT-15 μm vs. DWCNT-1.5 μm and MWCNT-7. ^e^ *p* < 0.05, MWCNT-7 vs. DWCNT-1.5 μm. ^f^ *p* < 0.05, DWCNT-15 μm vs. DWCNT-1.5 μm. ^g^ *p* < 0.001, MWCNT-7 vs. DWCNT-1.5 μm, 7 μm, and 15 μm.

### 3.8. CCL2, CCL3, and HO-1 in Non-Tumor Lung Tissue (Table 6)

CCL2, also known as Monocyte Chemoattractant Protein 1 (MCP-1), binds to chemokine receptors CCR2 and CCR4 and functions to attract and activate macrophages. CCL3, also known as monocyte inflammatory protein 1 alpha (MIP-1alpha), binds to chemokine receptors CCR1, CCR4, and CCR5 and functions to attract and activate macrophages and neutrophils and other lymphocytes. HO-1 is produced by macrophages as an anti-inflammatory mechanism [21]. Similarly to CD68-positive macrophages and the alveolar epithelial cell PCNA index, at the final sacrifice, the levels of CCL2 and HO-1 were increased with increasing DWCNT fiber length. In contrast, CCL3 was not increased in the lung tissues of any of the treated rats. None of these parameters were significantly elevated in the lungs of the MWCNT-7-treated rats at the final sacrifice (discussed below).

**Table 6:** Levels of inflammatory cytokines in non-tumor lung tissue at the final sacrifice ^$^.

	Group 1	Group 2	Group 3	Group 4	Group 5	Group 6
	Untreated	Vehicle	DWCNT			MWCNT-7
			1.5 μm (0.050 ^#^)	7 μm (0.232 ^#^)	15 μm (0.504 ^#^)	
CCL2 pg/mg protein lung tissue extract Mean ± SD	0.06 ± 0.01	0.07 ± 0.01	0.09 ± 0.03	0.13 ± 0.04 **	0.21 ± 0.04 ***	0.10 ± 0.03
CCL3 pg/mg protein lung tissue extract Mean ± SD	0.24 ± 0.05	0.21 ± 0.05	0.22 ± 0.03	0.21 ± 0.02	0.14 ± 0.03	0.15 ± 0.02
HO-1 pg/mg protein lung tissue extract Mean ± SD	0.25 ± 0.22	0.194 ± 0.11	0.17 ± 0.06	0.44 ± 0.03 *	0.62 ± 0.27 *	0.11 ± 0.06

^$^ Five animals were examined in each group. ^#^ Total fiber dose (mg)/rat. *, **, *** *p* < 0.05, 0.01, 0.001 vs. vehicle, respectively.

### 3.9. Fiber Retention in the Lungs

**Figure 10:**
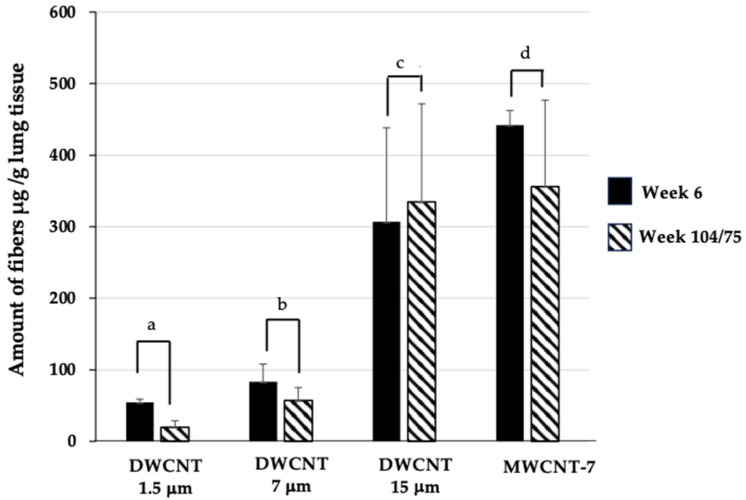
Comparison of retention rates of the three DWCNTs and MWCNT-7 in the lung at weeks 6 and 104 for DWCNTs and weeks 6 and 75 for MWCNT-7. The residual amounts of DWCNTs and MWCNT-7 in the lung tissue after 104 and 75 weeks are compared with those 6 weeks after administration. The results show increasing retention of fibers with increasing fiber agglomerate size (Table 1): (a) DWCNT-1.5 μm, (b) (37%); DWCNT-7 μm, (c) (68%); DWCNT-15 μm, (d) (100%); and MWCNT-7, (80%).

The amounts (μg/g lung tissue) of the three different DWCNTs and MWCNT-7 were measured at week 6 and week 104 for DWCNTs and week 6 and week 75 for MWCNT-7 (Figure 10). The results indicate that biopersistance of the DWCNT fibers in the lung increased with increasing length of the fibers.

## 4. Discussion

In the present study, we administered DWCNTs of different lengths into the lungs of rats using TIPS: 1.5 µm (D1.5), 7 µm (D7), and 15 µm (D15). Each of these groups of rats was administered approximately 22 × 10^12^ fibers per rat: 0.0504 mg per rat for D1.5, 0.232 mg per rat for D7, and 0.504 mg per rat for D15. All three lengths of DWCNTs induced lung toxicity. Overall, the DWCNTs were mildly carcinogenic in the rat lung. All lung tumors in the DWCNT-administered groups were bronchioloalveolar carcinomas. A single rat developed a visceral mesothelioma, discussed below. No other toxicities were associated with administration of these DWCNTs into the rat lung.

One rat in the D15 group developed mesothelioma on the visceral pleura. This is significant compared to the incidence of spontaneous mesothelioma in male Fischer 344 rats, with 22 cases of spontaneous mesothelioma in 51,326 male rats [22]. However, as discussed in Saleh et al. 2022 [9], development of visceral mesothelioma in rats after administration of CNTs into the lung is not necessarily related to the development of mesotheliomas in humans. An important difference in the visceral pleural in rats and humans is that the visceral pleura in rats is thin, composed of a pleural mesothelial layer and a basement membrane overlying the alveoli (see Figure 6 in [9] Saleh et al.), while in humans the visceral pleural mesothelium is separated from lung alveolar cells by a substantial layer of connective tissue. Consequently, events occurring in the lung alveoli adjacent to the visceral pleura could affect visceral pleural mesothelial cells in rats but would not affect visceral pleural mesothelial cells in humans. Notably, They found that HMGB1 was present in the pleural lavage of rats treated with MWCNT-7, a rigid MWCNT, but not in rats treated with DWCNT, and the lack of HMGB1 in the pleural cavity was also observed in the DWCNT-treated rats that developed mesothelioma [9]. Since thick, rigid MWCNTs such as MWCNT-7 are cytotoxic to mesothelial cells [15,16,23], and since damaged mesothelial cells release HMGB1, and HMBG1 is involved in the development of mesothelioma [12,13,14,24,25], the mechanism of MWCNT-7 induction of mesothelioma is relevant to humans. In contrast, in the absence of the demonstration of mesothelial cell cytotoxicity and release of HMBG1, the mechanism of DWCNT induction of visceral mesothelioma is likely to occur as a result of events in the alveoli and is not relevant to humans [9]. While the rat that developed mesothelioma in our study was found dead, and consequently could not be assessed for the presence of HMGB1 in the pleural cavity, the presence of a single visceral mesothelioma cannot be taken as evidence that administration of D15 into the lung resulted in translocation of D15 fibers into the pleural cavity and induction of mesothelioma by a mechanism relevant to humans.

In the MWCNT-7-treated group, 11 rats died from malignant mesothelioma, which is similar to the results of our previous studies and agrees with the cytotoxicity and pleural malignancy of MWCNT-7 administered into the rat lung, discussed above. The four rats that did not die from malignant mesothelioma were examined for the development of proliferative lung lesions. One rat developed bronchioloalveolar hyperplasia, and two rats developed bronchioloalveolar carcinoma. None of the four rats developed bronchioloalveolar adenoma. These results are compatible with the study by Hojo et al. who administered MWCNT-7 to rats using TIPS once every 4 weeks over the course of 2 years [8]. In their study 6 of 28 rats developed bronchioloalveolar carcinoma, 4 of 28 rats developed bronchioloalveolar adenoma, and 1 rat developed non-keratinizing epithelioma. In addition, the MWCNT-7-treated rats had increased granuloma counts, increased numbers of CD68 macrophages, and increased alveolar epithelial cell PCNA index at 6 weeks and at the final sacrifice. Thus, the carcinomas that developed in this group can be considered to be typical of bronchioloalveolar carcinomas induced by MWCNT-7.

It is notable that the incidence of lung tumor development was not lower in the D1.5 group compared to the D15 group. While the same number of fibers were administered to the rats in the D1.5, D7, and D15 groups, the different lengths of the fibers resulted in substantial differences in the total amount of material administered. Rats in the D1.5 group were administered 0.0504 mg DWCNT, rats in the D7 group were administered 0.2323 mg DWCNT, and rats in the D15 group were administered 0.5040 mg DWCNT. At six weeks granulomas had developed in the lungs of all three groups, and the granulation tissue count increased in proportion to DWCNT fiber length. Thus, longer fibers, which means more material administered, caused the development of more granulation tissue than shorter fibers. The alveolar epithelial cell PCNA index also increased with increasing fiber length; however, the increases were less than the increases in granulation tissue count. In contrast to the increases in granulation tissue count and alveolar epithelial cell PCNA index, CD68-positive macrophage count did not increase with increasing fiber length. These findings at six weeks indicate that sequestration of fibers in granulation tissue dampened the biological reaction to the fibers and may have lessened the difference in the reaction to shorter versus longer fibers.

At the final sacrifice, in addition to increases in granuloma count, increases in CD68-positive macrophages, CCL2, the alveolar epithelial cell PCNA index, and HO-1, which is involved in the generation of antioxidants, were found. All five parameters increased with increasing fiber length. These findings indicate that although the fibers were being sequestered in granulation tissue, DWCNT fibers were continuing to elicit a biological response. Thus, at 104 weeks, the difference in biological responses elicited by longer fibers versus shorter fibers was not proportional to the length of the fibers, likely due to sequestration of the fibers in granulation tissue; however, longer fibers appeared to elicit a stronger biological response compared to the shorter fibers.

Thus, at both 6 weeks and 104 weeks, the longer fibers appeared to elicit a stronger biological response compared to the shorter fibers. In addition, bronchioloalveolar hyperplasia was found in the lungs of 5 of 8 rats in the D7 group and 5 of 10 rats in the D15 group, but no bronchioloalveolar hyperplasia was found in any of the 12 rats in the D1.5 group (Table 4). However, the shorter fibers appeared to elicit a stronger carcinogenic response in the lung compared to the longer fibers with bronchioloalveolar carcinoma developing in the lungs of 4 of 12 rats in the D1 group, 3 of 8 rats in the D7 group, and 2 of 10 rats in the D15 group (Table 4). While there was no statistical difference in the incidence of carcinomas in the three groups, the increases in granulation tissue count, CD68-positive macrophages, CCL2, the alveolar epithelial cell PCNA index, HO-1, and hyperplasia in the D15 group compared with the D1.5 group was not reflected in the development of lung tumors in these groups.

The most obvious explanation for the discrepancy in carcinogenicity versus administered DWCNT dose is the low number of rats administered the three different DWCNTs and administration of only a single dose of each of the three different DWCNTs to the rats. This limitation in the present study was due to the fact that we used DWCNT of precise lengths specifically manufactured for our study, resulting in very limited quantities of materials available for the study.

A biologically relevant explanation for the observed pattern of biological responses observed in the present study is blockade of the smaller airways and bronchioles by the larger agglomerates formed by the longer fibers. This would result in decreased deposition of the longer fibers in the alveoli and decreased carcinogenicity elicited by the longer fibers. As shown in Table 4, administration of 0.0504 mg DWCNT 1.5 resulted in induction of four adenocarcinomas in 12 rats, administration of 0.2323 mg DWCNT 7 resulted in induction of three adenocarcinomas in 8 rats, and administration of 0.5040 mg DWCNT 1.5 resulted in induction of two adenocarcinomas in 10 rats. This corresponds to 6.6 carcinomas per rat per mg D1.5, 1.6 carcinomas per rat per mg D7, and 0.4 carcinomas per rat per mg D15. Decreased deposition of fibers in the alveoli would also result in decreased recruitment of macrophages per mg fiber into the alveoli (see Table 2). Importantly, in addition to decreasing deposition of fibers in the alveoli, blockade of the smaller airways and bronchioles would also inhibit movement of macrophages containing fibers that did reach the alveoli out of the alveoli to the mucociliary escalator. Restricted movement of macrophages with phagocytosed fibers would result in increased formation of granulation tissue in the lungs of rats administered longer fibers (see Table 2). In addition, prior to the formation of protective granulomas, restricted movement of macrophages with phagocytosed fibers out of the alveoli would result in damage to alveolar epithelial cells mediated by the response of macrophages to phagocytosed fibers, and subsequent tissue repair would result in the increased alveolar cell PCNA index observed in the lungs of rats administered longer fibers (see Table 2). However, the presence of CD68-positive alveolar macrophages, the alveolar epithelial cell PCNA index, CCL2, HO-1, and hyperplasia at two years was increased in the D7 and D15 groups compared with the D1.5 group (see Table 4, Table 5 and Table 6); these biological responses were not reflected in the development of lung tumors in these groups (see Table 4), indicating that other data needs to be considered.

An important point is the development of bronchioloalveolar hyperplasia (BAH) in the D7 and D15 groups. In the D7 group, three rats developed carcinomas, and the other five rats developed BAH. In the D15 group, two rats developed carcinomas, and five of the remaining rats developed BAH. Thus, the incidence of proliferative lung lesions in the three groups was 4 of 12 rats in the D1 group, 8 of 8 rats in the D7 group, and 7 of 10 rats in the D15 group. Consequently, potential development of lung tumors increased with increasing DWCNT fiber length. This result agrees with the increase in CD68-positive lung macrophages and the increase in PCNA positive alveolar epithelial cells with increasing fiber length at week 104 (Table 5).

Another interesting possibility that there were differences in the molecular signatures of one bronchioloalveolar carcinoma that developed in a D1.5 rat and one bronchioloalveolar carcinoma that developed in a D7 rat. This suggests the possibility that the agglomerates formed by DWCNT fibers of different lengths may have different effects on carcinogenic pathways. One possible mechanism would be different effects on macrophages and the production of tissue-damaging ROS and other cytotoxic and DNA-damaging molecules as the macrophages attempt to destroy the alveoli-invading pathogens/fibers, and different effects on the production of chemokines and inflammatory cytokines. One possible mechanism by which shorter and longer fibers could interact differently with macrophages is by forming smaller and larger agglomerates, with the larger agglomerates exerting a more cytotoxic effect on the engulfing macrophage. This in turn would result in exposure of alveolar cells to increased levels of intracellular cytotoxic molecules produced by macrophages when macrophages interact with larger agglomerates, resulting in alveolar cell damage and increased alveolar cell proliferation to repair the damage. Such a mechanism could lead to the findings shown in Table 2. At week 6 the levels of CD68-positive macrophages was similar in the D1.5 and D15 groups, while the D15 group had a markedly higher alveolar epithelial cell PCNA index compared to the D1.5 group.

Using flexible MWCNTs that formed tangled agglomerates, two in vitro studies by Sweeney et al., 2014 [26] and 2015 [27], reported that larger agglomerates formed by longer, flexible MWCNT fibers were more cytotoxic to macrophages than smaller agglomerates formed by shorter, flexible fibers; the agglomerates formed by the different-length MWCNTs are shown in Figure 1 in [26]. In our study, decreased macrophage cytotoxicity by the smaller agglomerates formed by the shorter DWCNT fibers could result in decreased macrophage cell death coupled with higher levels of secreted chemoattractants, resulting in relatively higher recruitment of macrophages into the lung in response to short fibers compared to longer fibers. The similar levels of CD68-positive lung macrophages in rats treated with short fibers (low fiber dose) and long fibers (high fiber dose), shown in Table 2, could be the result of such an effect by smaller and larger fiber agglomerates on engulfing macrophages.

In addition, the in vitro study by Sweeney et al. (2014) [26] also reported that the smaller aggregates formed by the shorter MWCNTs interacted with primary human pulmonary alveolar epithelial cells to a much greater extent than the larger agglomerates formed by the longer MWCNTs.

It is also notable that at the final sacrifice, the levels of CCL2, CCL3, and HO-1 were not significantly increased in the MWCNT-7 group. This would appear to be due to the level of CD68-positive macrophages in the lungs of these rats at the final sacrifice: the D1.5-treated rats and the MWCNT-7-treated rats had similar levels of CD68-positive macrophages (Table 5) and similar levels of CCL2, CCL3, and HO-1 (Table 6) at the final sacrifice. Thus, the lower levels of CD68-positive macrophages in the lungs of the D1.5 and MWCNT-7 compared to the D7 and D15 groups would result in lower levels of CCL2, which is secreted by macrophages, and lower levels of HO-1, which is produced by macrophages as an anti-inflammatory mechanism [21]. This finding is in agreement with those of Saleh et al. [9]. They reported that while the levels of CCL2 were significantly elevated in the lungs of MWCNT-7-treated rats compared to the controls, the CCL2 levels were approximately 50% of the levels in the lungs of rats treated with 0.5 mg DWCNT [9]. These findings all suggest relatively low levels of inflammation in the lungs of rats approximately 1 year after administration of MWCNT-7. Importantly, lower levels of inflammation in the lungs of rats exposed to D1.5 and MWCNT-7 compared to D7 and D15 do not contradict the findings that the molecular signature of the carcinoma that developed in the MWCNT-7-treated rat (with lower inflammation) is more similar to the signature of the carcinoma that developed in the D7-treated rat (with higher inflammation) than the signature of the carcinoma that developed in the D1.5-treated rat (with lower inflammation). Taken altogether, these differing findings suggest that in addition to induction of the production of cytokines and chemokines by macrophages, fiber flexibility/rigidity, fiber length, and the particulates/agglomerates formed by the fibers can stimulate alternative tumorigenic processes. As suggested above, one possible mechanism by which an alternative tumorigenic process could be stimulated by different fibers is macrophage cytotoxicity and exposure of alveolar epithelial cells to macrophage intracellular cytotoxic and DNA-damaging/mutagenic agents. Cytotoxicity of mesothelial cells plays an essential role in fiber-induced mesothelioma [11,15]. While the role of cytotoxicity in the development of fiber-induced mesothelioma is not equivalent to the proposed role of macrophage cytotoxicity in the development of fiber-induced lung tumors, in both types of tumor promoting pathways, fiber-induced cytotoxicity activates pathways that are not activated by fibers that are not cytotoxic. The propositions discussed above should be investigated in future studies.

The increased levels of CD68-positive alveolar macrophages, the alveolar epithelial cell PCNA index, CCL2, HO-1, and hyperplasia at two years coupled with lower carcinogenicity per mg fiber in the D7 and D15 groups compared with the D1.5 group can also be explained by blockade of smaller airways and bronchioles by the aggregates formed by longer DWCNT fibers. As discussed above, blockade of smaller airways and bronchioles would result in deceased deposition of longer fibers in the alveoli coupled with decreased clearance of fibers via the mucociliary escalator. This would result in exposure to lower levels of fibers but for an increased period of time. Consequently, lung carcinogenesis in the D7 and D15 groups would be delayed compared to the D1.5 group. In addition, increased clearance of D1.5 fibers would result in decreased activity in response to these fibers. Therefore, at two years, the longer fibers would induce increased biological activity coupled with fewer fully developed tumors. In addition, macrophage cytotoxicity could also play a role the carcinogenic potential of DWCNT fibers of different lengths. Overall, the differences in carcinogenicity of the DWCNTs used in the present study can be explained by blockade of smaller airways and bronchioles, possibly in conjunction with increased macrophage cytotoxicity, and by the agglomerates formed by longer fibers; however, this premise needs to be verified in future studies.

The major limitation of this study is the small number of rats administered each DWCNT. As noted above, this was due to the fact that we used DWCNTs of precise lengths specifically manufactured for our study, resulting in very limited quantities of materials available for the study. Despite this constraint, our findings are consistent with previous results demonstrating that double-walled carbon nanotubes (DWCNTs) are carcinogenic in rat lungs.

Furthermore, the data suggest that the size and morphology of fiber agglomerates may influence carcinogenic potential. This highlights the importance of considering not only the intrinsic properties of carbon nanotubes, such as length and wall number, but also the physical form in which they are encountered. When evaluating the potential human health risks of CNT exposure, the characteristics of airborne agglomerates in occupational or environmental settings should be carefully taken into account.

## Figures and Tables

**Table 1:** Size of three DWCNTs and MWCNT-7 shortly before dosing.

	DWCNT 1.5 μm	DWCNT 7 μm	DWCNT 15 μm	MWCNT-7
Length of the agglomerates	4.5 ± 2.2 μm	6.8 ± 8.2 μm	10.2 ± 5.8 μm	8.5 ± 4.5 μm
Diameter of the agglomerates	143.7 ± 120.1 nm	268.2 ± 416.1 nm	227.6 ± 172.5 nm	63.9 ± 20.0 nm

All the DWCNT fibers form irregular agglomerates. At least 100 agglomerates formed by each of the fibers were measured. The lengths of the DWCNT agglomerates increase with increasing fiber length.

## Data Availability

The original contributions presented in this study are included in the article. Further inquiries can be directed to the corresponding authors.

## Abbreviations

The following abbreviations are used in this manuscript:

DWCNT	Double-walled carbon nanotube
MWCNT	Multi-walled carbon nanotube
TIPS	Trans-tracheal intra-pulmonary spraying
CNT	Carbon nanotube
HMGB1	High mobility group box protein 1
PCNA	Proliferating cell nuclear antigen
SEM	Scanning electron microscope
TEM	Transmission electron microscope
