# Correction: Ahmed et al. Comparative Carcinogenicity of Double-Walled Carbon Nanotubes of Different Lengths Administered by Intratracheal Installation into Rat Lungs. *Nanomaterials* 2025, *15*, 1402

**DOI:** 10.3390/nano16130808

**Published:** 2026-06-30

**Authors:** Omnia Hosny Mohamed Ahmed, Dina Mourad Saleh, William T. Alexander, Hiroshi Takase, Yuhji Taquahashi, Motoki Hojo, Ai Maeno, Katsumi Fukamachi, Min Gi, Akihiko Hirose, Shuji Tsuruoka, Satoru Takahashi, Hiroyuki Tsuda, Aya Naiki-Ito

**Affiliations:** 1 Nanotoxicology Project, Nagoya City University, Nagoya 467-8603, Japan; drdina@aun.edu.eg (D.M.S.); william@phar.nagoya-cu.ac.jp (W.T.A.); htsuda@phar.nagoya-cu.ac.jp (H.T.); 2 Department of Experimental Pathology and Tumor Biology, Graduate School of Medical Sciences, Nagoya City University, Nagoya 461-8601, Japan; sattak@med.nagoya-cu.ac.jp; 3 Department of Forensic Medicine and Clinical Toxicology, Faculty of Medicine, Aswan University, Aswan 81528, Egypt; 4 Department of Forensic Medicine and Clinical Toxicology, Faculty of Medicine, Assiut University, Assiut 71515, Egypt; 5 Core Laboratory, Graduate School of Medical Sciences, Nagoya City University, Nagoya 461-8601, Japan; takase@med.nagoya-cu.ac.jp; 6 Division of Cellular and Molecular Toxicology, Center for Biological Safety & Research, National Institute of Health Sciences, Kawasaki 210-0821, Japan; taquahashi@nihs.go.jp; 7 Department of Pharmaceutical and Environmental Sciences, Tokyo Metropolitan Institute of Public Health, Tokyo 169-0073, Japan; motoki_hojo@member.metro.tokyo.jp (M.H.); ai_maeno@member.metro.tokyo.jp (A.M.); 8 Department of Neurotoxicology, Graduate School of Medical Sciences, Nagoya City University, Nagoya 461-8601, Japan; kfukamac@med.nagoya-cu.ac.jp; 9 Department of Environmental Risk Assessment, Graduate School of Medicine, Osaka Metropolitan University, Osaka 545-8585, Japan; mwei@omu.ac.jp; 10 Chemicals Evaluation and Research Institute (CERI), Tokyo 112-0004, Japan; akihikoh@dranihs.net; 11 Research Institute for Supra-Materials, Shinshu University, Nagano 380-8553, Japan; sh.tsuruoka@neura.co.jp

In the original publication [1], there was a mistake in the assembly of Figure 4. The image panel for Figure 4B (Vehicle group) was inadvertently derived from the same source image as Figure 4A (Untreated group) during the trimming process, resulting in an unintended overlap between Figure 4A,B. 

The correct Figure 4 appears below:

**Figure 4:**
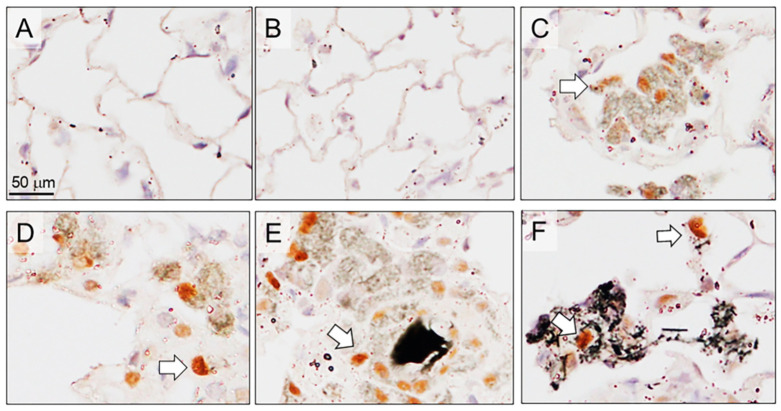
PCNA-positive macrophages (brown nuclei) in the alveoli at week 6. (**A**) Untreated. (**B**) Vehicle. (**C**) DWCNT 1.5 μm. (**D**) DWCNT 7 μm. (**E**) DWCNT 15 μm. (**F**) MWCNT-7. Lung sections of rats administered DWCNTs at 6 weeks. Among the PCNA-positive macrophages that compose the granulation tissue, some clearly phagocytose DWCNTs and MWCNT-7 (arrows) (**C**–**F**).

The authors state that the scientific conclusions are unaffected. This correction was approved by the Academic Editor. The original publication has also been updated.
